# Aiming Higher to Test a Bend in the Curve of Biodiversity Loss: The Challenge of Halt‐The‐Loss Targets

**DOI:** 10.1002/ece3.73157

**Published:** 2026-02-25

**Authors:** Mairenn C. Attwood, Richard D. Gregory, Nick J. B. Isaac, Fiona Burns

**Affiliations:** ^1^ Department of Zoology University of Cambridge Cambridge UK; ^2^ FitzPatrick Institute of African Ornithology University of Cape Town Cape Town South Africa; ^3^ RSPB Centre for Conservation Science The David Attenborough Building Cambridge UK; ^4^ Centre for Biodiversity and Environment Research, Department of Genetics, Evolution and Environment University College London London UK; ^5^ UK Centre for Ecology & Hydrology Wallingford Oxfordshire UK

**Keywords:** biodiversity indicators, biodiversity policy, biodiversity recovery, biodiversity targets, Convention on Biological Diversity, Global Biodiversity Framework

## Abstract

Efforts to ‘bend the curve’ of biodiversity loss involve setting targets to halt declines. Here, we propose an empirical test of such targets. This test states the probability that the rate of change in a biodiversity indicator is greater than or equal to zero. We used a combination of real and simulated data to explore factors affecting test performance. We found that while smoothing had a minimal effect, the outcome depends heavily on the variability and number of species in a dataset. This suggests that thresholds for target acceptance should be set on a case‐by‐case basis. Adding data for subsequent years could retrospectively change the outcome in a target year. Assessments made with data only up until the target year should therefore be regarded as interim, with the assessment of the target only finalised once subsequent data are also included. We recommend that simulations are used a priori to choose smoothing levels and to set thresholds for accepting a target has been met.

## Graphical abstract

We present a test to evaluate targets to ‘halt the loss’ of biodiversity. We use simulations to explore the accuracy of this test with different datasets. We recommend that thresholds for target assessments are set on a case‐by‐case basis using simulations, and that assessments are regarded as interim until data following the target year can be included.

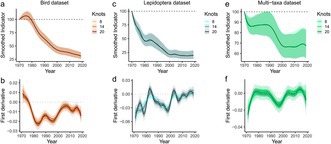

## 1 Introduction

In the face of the biodiversity crisis, there are concerted efforts to stem and reverse losses. These efforts involve targets at regional, national and international levels, which have become increasingly specific over time. Targets can be categorised as either action targets (e.g., the Global Biodiversity Framework), which focus on the drivers of biodiversity loss; or outcome targets, which focus on the state of biodiversity. Outcome targets exist on a continuum of ambition: from ‘slow‐the‐rate‐of‐loss’, to ‘halt‐the‐loss’ and ‘nature positive’ (Mace et al. 2018). A major ‘slow‐the‐rate‐of‐loss’ target was adopted at the 2002 Convention on Biological Diversity (Balmford et al. 2005), with more recent targets now focussing on the latter two categories. ‘Halt‐the‐loss’ targets aim to stabilise declines in biodiversity (Leadley et al. 2022; The Environmental Targets (Biodiversity) (England) 2023; Geldmann et al. 2023; European Union Nature Restoration Law 2024; United Nations Environment Programme 2023), while ‘nature positive’ targets aim for net increases in biodiversity indicators relative to some baseline (Bull et al. 2020; Milner‐Gulland 2022). Both imply an upwards bending of the curve of biodiversity loss. With declining trends globally, ‘halt‐the‐loss’ targets are a necessary first step to achieving ‘nature positive’, and we need accurate assessments of both to create accountability and inform progress.

Evaluating outcome‐based targets is challenging because uncertainty in overall trends makes it difficult to detect significant changes, particularly at 95% confidence intervals or over short timeframes (Johnson et al. 2024; Leung and Gonzalez 2024). Timeframes are often short to provide the impetus for urgent action (IUCN 2008), and targets may even stipulate comparisons between adjacent years to determine success (e.g., The Environmental Targets (Biodiversity) (England) 2023).

‘Halt‐the‐loss’ targets are particularly difficult to assess because they can be met with a stable trend, which is difficult to detect using null‐hypothesis statistical tests (the default option for biodiversity indicators). Whereas a target for an increasing trend implies a test against the null hypothesis of no change, a ‘halt‐the‐loss’ target cannot be assessed this way (Dixon and Pechmann 2005; Schmidt and Meyer 2008; Smith 2020). This is because failure to reject the null hypothesis (of no significant change in the indicator) does not mean the null hypothesis can be accepted (Smith 2020). One way to overcome this problem is to move away from binary frequentist criterion (e.g., alpha = 0.05) to a probabilistic assessment.

Here, we use the example of a multi‐species abundance indicator to illustrate the general principles of assessing a ‘halt‐the‐loss’ target. We present a ‘first derivative’ test based on the probability that the rate of change in an indicator is at or above zero in a given year. This probability is given by Bayesian credible intervals, which are intuitive to understand and useful when uncertainty is high (Wade 2000; Brooks et al. 2008; Gerrodette 2011). This emulates the risk framework recommended by Leung and Gonzalez (2024) and circumvents issues with null hypothesis testing: rather than relying on 95% as the default cut‐off in certainty (which runs a high risk of a false negative error), the threshold to accept that the target has been met is explicitly linked to the risk of both false positive and false negative errors, informed by the dataset at hand.

A suite of factors might affect the ability of this (and, indeed, any) test to detect a halt in species abundance decline. These include the following: (1) how closely individuals sampled in time‐series datasets reflect the true population; (2) the number of species in the indicator; (3) noise in the individual species time‐series (‘trend variability’), composed of inter‐annual and inter‐specific variation, plus measurement error of trends; (4) the strength of the underlying signal to be detected—whether the growth rate is stable, changing slightly or strongly; (5) the number of years with a particular trend; and (6) the level of smoothing applied. Factors 1–4 are properties of the dataset used to construct the indicator, while 5 is affected by the target date and when the outcome is reported. Factor 6 (smoothing) is chosen during indicator construction.

Smoothing in multi‐species indicators is used to reduce the impact of year‐to‐year fluctuations (e.g., due to varying weather conditions) and reveal the underlying trend. However, it can be difficult to choose the appropriate level of smoothing (Fewster et al. 2000; Eaton et al. 2015; see Figure S1), particularly as this can affect the trade‐off between type I (false positive) and type II (false negative) errors. When controlled within a GAM or thin‐plate spline, the convention is to set the degrees of freedom to 0.3 × years of data (based on Fewster et al. 2000) (e.g., Amano et al. 2012; Nash et al. 2013; Wright et al. 2014; Dambly et al. 2021). However, the appropriate level of smoothing will vary with particular objectives and datasets, and *should* be set on a case‐by‐case basis (Fewster et al. 2000).

Additional years of data could also affect the first derivative of the indicator. Targets are often set with reporting dates close to the target year; for example, the 2030 England Species Abundance Target has a reporting date of 15 April 2032, by which time only data to 2030 will realistically be included (as this requires compilation and analysis of multiple monitoring programmes). However, the value in the final year of a smoothed indicator is subject to change when subsequent years' data are added (Freeman et al. 2001; Gregory et al. 2006; Eaton and Noble 2023).

To investigate the effect of these factors (2–6) on the first derivative test that we propose using to assess a ‘halt‐the‐loss’ target, we trialled the test on three datasets which combine real and simulated data.

## 2 Methods

We simulated species time‐series with known population growth rates and appended these to real species' abundance data. We then fitted a multi‐species indicator with three levels of smoothing and calculated the probability that the first derivative of the indicator was ≥ 0 in each year. We fitted the multi‐species indicators using the Freeman method, which is based on the geometric mean of individual species' time‐series (Freeman et al. 2020; Bane et al. 2023).

**FIGURE 1:**
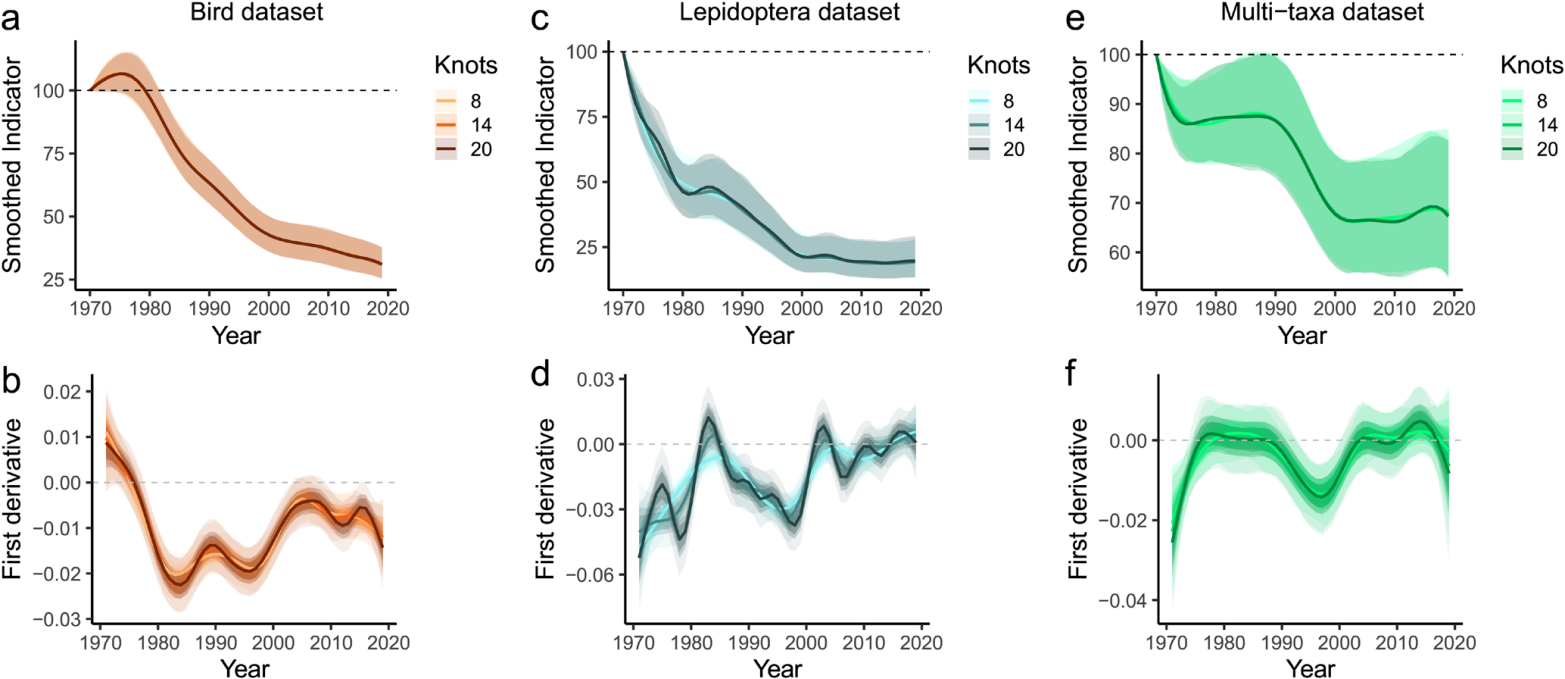
Multi‐species indicators (using the Freeman method) (top row) and associated first derivatives (bottom row) for the three datasets, before appending simulated data. (a) the bird dataset, showing very little differentiation with different levels of smoothing (knots 8–20), although (b) slight variation can be seen across knot values in the first derivative (i.e., rate of change). The first derivative of the indicator remains below zero for all knot numbers from mid‐1970 onwards. (c) The Lepidoptera dataset, showing a similar decline as the birds, though there is some stabilisation towards the end of the time series. (d) The associated first derivative, with higher numbers of knots (e.g., 20) showing more fluctuations than a lower number (e.g., 8). Here, using the first derivative to test for a halt in abundance decline would have detected halts in the 1980s and 2000s with 20 knots, but not with 8 knots. (e) The multi‐taxa dataset, showing slower overall declines, and the associated first derivative in (f), where a decline is only detected periodically.

We compared three species abundance datasets with contrasting properties. (1) 39 England priority bird species (‘bird dataset’); (2) 39 England priority butterfly and moth species (‘Lepidoptera dataset’, Botham et al. 2022; Harrower et al. 2025); and (3) 1132 species from a range of taxa (‘multi‐taxa dataset’) (details in Table S1). Before appending the simulated data, the bird dataset showed strong and sustained evidence for a decrease from 1970, while declines in the Lepidoptera and multi‐taxa datasets appeared to have somewhat stabilised by 2019 (Figure 1). The first two datasets contain the same number of species but differ in interannual and interspecific variability—the Lepidoptera dataset is ‘noisier’ than the bird dataset. This allows us to compare how trend variability affects the test outcome, under simulated trends (all expressed as per annum) of 3% (strong increase), 1% (increase) and −1% (decrease). We expected the greater number of species in the multi‐taxa dataset to enable more accurate trend detection (and this provides a more realistic example for testing a national biodiversity target; these are a subset of species in the England species abundance indicator; DEFRA 2024). We therefore set this dataset a more stringent test—detecting a 2%, 0.1% (close to stability) and −1% simulated (per annum) change in growth rate.

For each dataset, we simulated 8 years of time series abundance data for individual species, using the ‘simulate_indicator’ function from the BRCindicators package (v.1.3.13) (August et al. 2021). This sets an underlying growth rate, plus variability around this rate at interspecific, interannual and ‘measurement error’ levels (values and methods to calculate these in Table S2). We joined these simulated time series individually to the real data (matching the abundances from 2019). We then pre‐smoothed the resultant time series following the England Species Abundance Index methodology (using a thin plate spline with knots set as 0.3 × timespan; DEFRA 2024), and combined the time series into multi‐species indicators (Freeman et al. 2020). Smoothing is controlled by the number of knots in the indicator; more knots allow for more fluctuations (i.e., a less smooth trend). We trialled each indicator with 8, 14 and 20 knots (0.14 × timespan, 0.25 × timespan and 0.35 × timespan respectively; chosen based on exploratory trials with values above and below the conventional 0.3 × timespan), and repeated this process for 50 runs of simulated data.

For each set of 50 simulations, we present the average probability that the first derivative (equivalent to logLambda in the Freeman model) is ≥ 0 in each year, that is, the proportion of the posterior distribution that lies above zero. This is the probability that the multi‐species indicator is stable or increasing between any 2 years, indicating that a target to halt species decline has been successfully met. Values above 50% indicate that an increasing trend is more likely than a decreasing trend. We also present frequency histograms of test outcomes in the final year of simulated data (plus example indicators and first derivative plots in Figure S2).

## 3 Results

**FIGURE 2:**
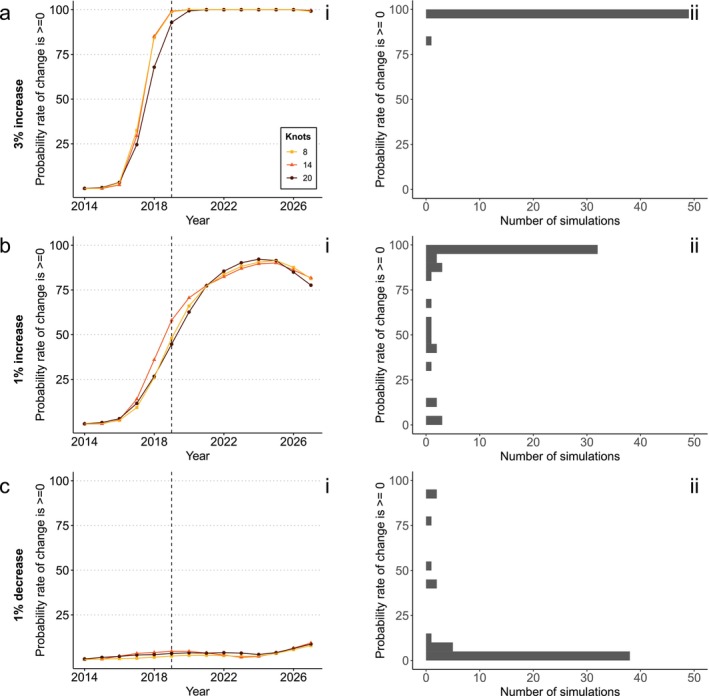
Outcomes of simulations and tests for the bird dataset given a simulated (a) 3% abundance increase, (b) 1% abundance increase and (c) 1% abundance decrease. Simulated years are to the right of the dotted lines (2019). (i) The average test results (probability that the first derivative of the indicator is ≥ 0) under three different levels of smoothing. (ii) The frequency distribution of test results in 2027 for 14 knots across simulation runs, grouped in bins of 5%.

With a simulated abundance increase of 3% in the bird dataset, the test averaged between 98%–100% probability that the rate of change was zero or positive (Figure 2ai). With a smaller simulated abundance increase of 1%, on average the test produced a lower probability of finding that the rate of change was ≥ 0 than for the 3% increase, though average values remained high (around 70%–95%; Figure 2aii). It also took longer (5 years with the 1% increase, compared to 1 year with the 3% increase) for the test to reach high probabilities of a rate of change ≥ 0.

The risk of a false positive error if abundance declines had continued was low (average test values between 1% and 16%) (Figure 2c). Changing the smoothing (number of knots) had minimal impact on the test results for any of the three simulated trends (Figure 2i).

**FIGURE 3:**
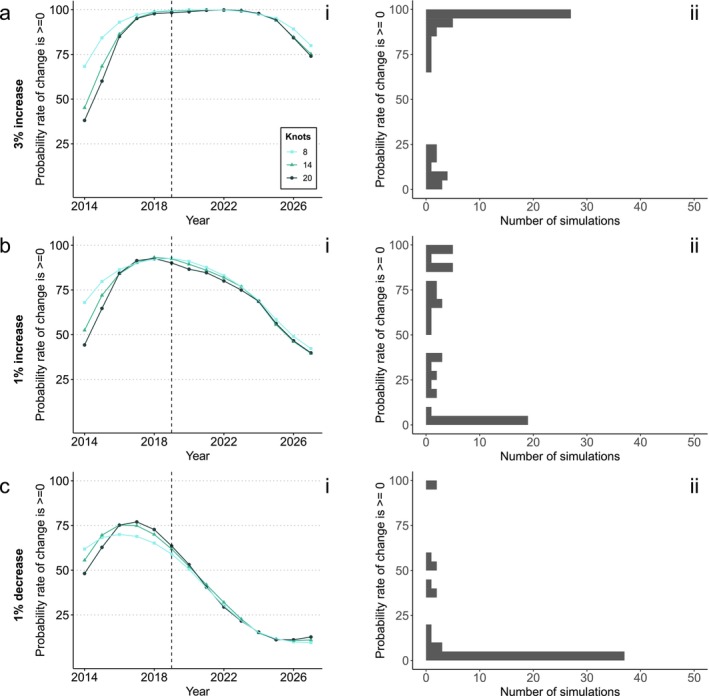
Outcomes of simulations and tests for the Lepidoptera dataset given a simulated (a) 3% abundance increase, (b) 1% abundance increase and (c) 1% abundance decrease. Simulated years are to the right of the dotted lines (2019). (i) The average test results (probability that the first derivative of the indicator is ≥ 0 in a given year) under three different levels of smoothing (knot numbers). (ii) The frequency distribution of test results in 2027 for 14 knots across simulation runs, grouped in bins of 5%.

By contrast, the Lepidoptera showed much lower consistency between simulations (Figure 3ii). Even with a 3% simulated increase, some simulations perceived a decline, likely due to the high interannual and interspecific variation around this trend. Results split bimodally, with the test either finding > 65% or < 25% probability that the decline had been halted (i.e., rate of change was ≥ 0). On average with the 3% increase, the probability of detecting a rate of change ≥ 0 ranged around 75%–100% (Figure 3ai).

With a 1% underlying increase, the average test value was between 40% and 90%, though the most common outcome was for 0%–5% probability that the rate of change was ≥ 0 (Figure 3b). With a 1% abundance decrease there was an average probability of around 10%–60% that the rate of change was zero or positive; the test gained greater accuracy with more years of data (Figure 3c). Changing the smoothing parameter had a bigger impact on the Lepidoptera indicator than the bird indicator, probably because of greater interannual variation in the Lepidoptera dataset (Figure 1). The test performed slightly better with the smoothest option (8 knots; Figure 3). However, the differences between smoothing levels remained relatively small (Figure 3a–c).

**FIGURE 4:**
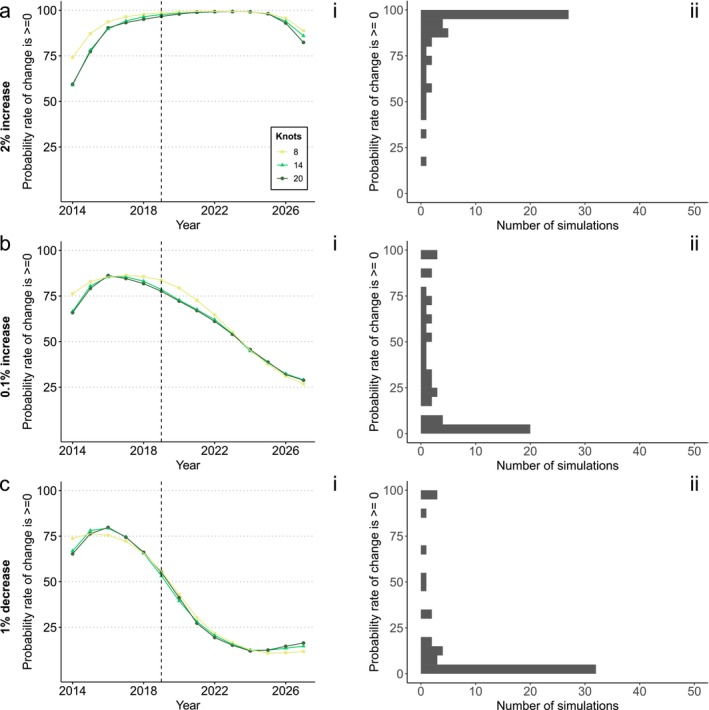
Outcomes of simulations and tests for the multi‐taxa dataset given a simulated (a) 2% abundance increase, (b) 0.1% abundance increase and (c) 1% abundance decrease. Simulated years are to the right of the dotted lines (2019). (i) The average test results (probability that the first derivative of the indicator is ≥ 0 in a given year) under three different levels of smoothing. (ii) The frequency distribution of test results in 2027 for 14 knots across simulation runs, grouped in bins of 5%.

For the multi‐taxa dataset, with a 2% increasing trend we found on average > 90% probability of a rate of change ≥ 0 over all but the last year (Figure 4ai). However, we found that a near‐stable trend (0.1% increase) would be difficult to detect (Figure 4bi). The average probability of a rate of change ≥ 0 ranged between 25% and 80% for this trend. Correctly finding a rate of change ≥ 0 was less likely with more years of data. For this near‐stable trend, the most common result in the final year was a 0%–5% probability of a rate of change ≥ 0 (Figure 4bii). This means the risk of a false negative error was high, even if a low threshold (e.g., at or below 60%) was accepted as meeting the target. In contrast, the risk of a false positive error was low with an underlying trend of −1% (Figure 4c).

**FIGURE 5:**
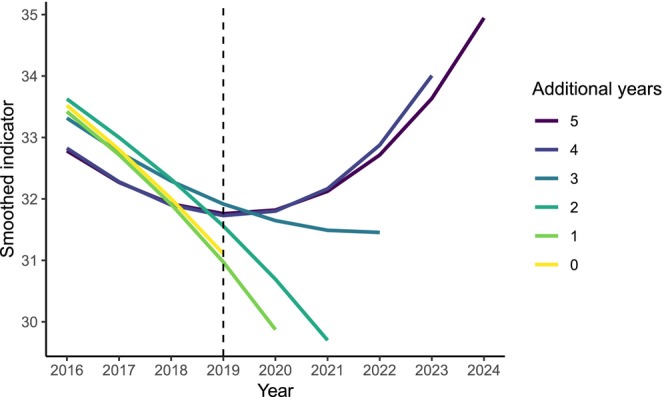
The impact of having additional years' data of a positive trend. This is based on the bird dataset with a 1% increasing growth rate in every simulated year (from 2019), and knots set as the total timespan × 0.17 to the nearest integer. Even though the data are identical in years 2019–2023 in every case, the indicator curve in 2019–2022 only bends to reflect the increase after 4 years of positive trend data. This is the point of smoothing—reducing short‐term trends—but demonstrates that subsequent years of data can qualitatively change the assessment from a given year.

Finally, we looked at the number of years' data with a positive trend that would be required to detect a ‘bending of the curve’. This depended on the dataset and level of smoothing, but for example required 4 years of data for the birds dataset with a +1% growth rate, assuming no interannual variation (Figure 5). If the target year was 2020, an assessment with the data up to 2020 or even 2022 would have found that the first derivative was negative (i.e., the target had not been met). This assessment for 2020 would have changed qualitatively in retrospect after including data from 2023 onwards. This issue is compounded by the credible interval expanding in the final year of data, which reduces certainty when data end in the test year (Figure S3).

## 4 Discussion

We show that the first derivative test could, with some care, be effectively used to assess ‘halt‐the‐loss’ targets, although assessments vary widely with the strength and variability of the underlying trend. Using a 95% threshold for accepting a decline has been halted would result in a high rate of false negative errors: there would be a strong risk of concluding the target was missed when in fact it was achieved. This concurs with previous work using indicators to test for trends (Johnson et al. 2024; Leung and Gonzalez 2024). There is no ‘one‐size‐fits‐all’ threshold alternative for accepting a decline has been halted. When there is minimal trend variability, a higher threshold strikes a better balance between false positive and false negative errors (as seen with the bird dataset, Figure 4) than for datasets with more trend variability (as seen in the Lepidoptera dataset, Figure 3).

In the Lepidoptera dataset, which is based on few species, with high variation in population growth rates, there is a serious risk of obtaining high confidence in a misleading value (Figure 3ii). With a 1% growth rate, a large proportion of simulations end up being very confident about the trend, but in the wrong direction (19 of 50 simulations end with a 0%–5% probability of a rate of change ≥ 0). It is likely Lepidopterans' life‐history (i.e., capacity for large fluctuations over short time periods), combined with the small number of species in the dataset that causes this issue, rather than the quality of the data. In such scenarios, great care would be needed to apply any statistical test of the target. We therefore advise that researchers test the consistency of their data between simulation runs—that is, plotting a histogram as shown in panels (ii) of Figures 2, 3, 4, as well as inspecting the indicators themselves (e.g., Figure S4)—before proceeding with a test. Code to do so is available alongside the Supporting Information.

Across the three datasets we used, the level of smoothing had minimal impact on the test outcome; however, this may not apply to other datasets. We recommend that any test of a ‘halt‐the‐loss’ target be informed by a simulation exercise similar to that described above. These insights should be used to determine the most appropriate level of smoothing for the test, which would be publicised in advance of the test date to maintain transparency and avoid criticism of possible bias.

We find two main challenges of using our approach to test ‘halt‐the‐loss’ targets. First, we are unlikely to be able to confidently conclude we have met the target unless we have overshot it. If the underlying growth rate is close to zero, then the proportion of the first derivative confidence interval ≥ 0 is likely to be around 50%. Even with a low burden of proof, say 60% probability, there remains a fair risk of both false positive and false negative errors. This challenge would apply to any test using similar datasets to those used here and would be further exacerbated under a standard significance test using alpha = 0.05 as a cut‐off (which would overwhelmingly produce false negative errors).

The best way to reduce errors is to improve the quality and quantity of raw data that feeds into the indicator: any model and test is only as good as the data that form it. Improving the raw data will affect the extent to which species trends reflect true change (e.g., due to spatial/temporal biases in data collection, site/surveyor turnover and changes in methods). Stable long‐term funding to secure and improve species monitoring therefore remains a priority (Gonzalez et al. 2023).

The risk of errors can also be reduced by including subsequent years of data. The exact number needed will depend on the dataset (emphasising the need for case‐by‐case simulations), but, for example, would be at least 3 years for the bird dataset (Figure 5).

Second, avoiding a consistent threshold for assessing targets creates a sense of ambiguity. This may be challenging to communicate to policymakers (Sarkki et al. 2014) and risks post hoc probabilities being chosen to state that a target has been met. To avoid this, thresholds should be set in advance of the test year, informed by simulating possible scenarios with the relevant historical data. Case‐by‐case simulations offer an adaptable approach to strike the balance between ambition and the constraints of uncertainty in the data being used (e.g., see simulations based on the Living Planet Index; McRae et al. 2025).

Taking a step back, policymakers and researchers should think critically about the scope of the target, what the datasets themselves represent and what meeting the target might mean: broad, multi‐taxa targets will ultimately only represent trends in taxa where there are available data, and underlying trends may vary between taxa and sites (Gregory and Van Strien 2010; Van Strien et al. 2012; Valdez et al. 2023) (contrasting with the assumption in our simulations that there is one true ‘underlying trend’). A statistical target to halt decline might be met in the fashion we describe and yet many species' populations might still be under severe decline. An ‘average’ increasing indicator trend could be achieved while many species are still declining, which brings into question whether the ultimate aim of ‘halting decline’ has been achieved. This is not to say that such tests are flawed but that subsequent and additional tests are likely to be required to have confidence that we achieved the desired outcomes. In our example a further test, beyond halting the average trend, might be to stipulate that no species was declining severely or some such (and set in place necessary definitions). Naturally, each of the frameworks we describe above propose multiple targets to capture different dimensions of biodiversity loss and recovery. Furthermore, designing assessments from the ground level should prioritise the drivers and actions needed to change the course of any declines in a holistic fashion (Gonzalez et al. 2026).

In conclusion, calculating the probability that the rate of change in an indicator is zero or positive is a promising way to test targets for halting biodiversity declines. Our results illustrate this for multi‐species abundance indicators, but we expect that with adaptation, they could be generalised to other metrics of biodiversity change (such as species richness or occupancy). Applying this test to real‐world datasets, we find that the thresholds for accepting a target has been met should be set on a case‐by‐case basis, depending on the number of species within and variability of the dataset at hand.

With high trend variability and few species, using this test runs a high risk of drawing an incorrect conclusion (with high confidence). In such cases, it may be inappropriate to test a target without revising the dataset. The focus instead should be to reduce bias and include more species and/or taxa. We recommend that simulations are run with the target‐relevant dataset to set both smoothing and acceptance thresholds. Accuracy in assessing targets increases (often drastically) with additional years of data following the target year, which should be considered when setting reporting dates. If data are only available up until the target year, the test may need to be considered an interim assessment and updated in subsequent years when further data are available.

## Author Contributions

**Mairenn C. Attwood:** conceptualization (equal), formal analysis (lead), writing – original draft (lead), writing – review and editing (equal). **Richard D. Gregory:** conceptualization (equal), methodology (equal), supervision (supporting), writing – review and editing (equal). **Nick J. B. Isaac:** conceptualization (equal), methodology (equal), software (lead), supervision (supporting), writing – review and editing (equal). **Fiona Burns:** conceptualization (equal), formal analysis (supporting), methodology (equal), supervision (lead), writing – review and editing (equal).

## Funding

This work was supported by the Natural Environment Research Council (NE/S007164/1), via an internship hosted by the Royal Society for the Protection of Birds (RSPB) at the Cambridge Conservation Initiative (CCI).

## Conflicts of Interest

The authors declare no conflicts of interest.

## Supporting information


**Data S1:** ece373157‐sup‐0001‐Supinfo.pdf.

## Data Availability

Code is available in Apollo: https://doi.org/10.17863/CAM.118293.
