# Supplementary material for: Aiming Higher to Test a Bend in the Curve of Biodiversity Loss: The Challenge of Halt‐The‐Loss Targets
Source: Ecol Evol. 2026 Feb 25;16(3):e73157. doi: 10.1002/ece3.73157 (PMC12935463; doi:10.1002/ece3.73157)
Supplement: Supplementary file 1 — Data S1: ece373157‐sup‐0001‐Supinfo.pdf. [file ECE3-16-e73157-s001.pdf]

# Supporting Information

Table S1: Details of the datasets and data owners for the multi-taxa dataset. The 'Bird' and 'Lepidoptera' datasets are subsets of the multi-taxa dataset, based on the England list of priority species (Natural Environmental and Rural Communities Act 2006 - Section 41). For each dataset, we removed species without abundance estimates in 2019 (total n = 10 species removed from the multi-taxa dataset, leaving the numbers shown in the table). The bird dataset totalled 39 species after removing 5 species without estimates in 2019. The 'Lepidoptera' dataset is based on the England list of priority butterfly species (n = 21), with 18 species added from the list of priority moth species, to create a total of 39 species (to be comparable to the bird dataset).

| Dataset origin | Data owner/partners | Taxon | No. of species in dataset | Timespan |
|---|---|---|---|---|
| **Breeding Bird Survey (BBS)** https://zenodo.org/records/14161736 | British Trust for Ornithology (BTO), Royal Society for the Protection of Birds (RSPB), Joint Nature Conservation Committee (JNCC) | Birds | 2 | 1970-2019 |
| **Rare Breeding Birds Panel (RBBP)** https://rbbp.org.uk/data-requests/ | BTO, RSPB, JNCC, Rare Breeding Birds Panel (RBBP) secretariat | Birds | 21 | 1970-2019 |
| **Seabird Monitoring Programme (SMP)** https://app.bto.org/seabirds/public/index.jsp | BTO, JNCC in association with RSPB | Birds | 10 | 1986-2019 |
| **Wintering Waterbird Indicator (WWBI)** Wild bird populations in the UK and England, 1970 to 2024 - GOV.UK | BTO, RSPB, JNCC, NatureScot (from Wetland Bird Survey (WeBS) and Goose and Swan Monitoring Programme) | Birds | 20 | 1975-2019 |
| **Wild Bird Indicator (WBI)** Wild bird populations in the UK and England, 1970 to 2024 - GOV.UK | Various* | Birds | 107 | 1970-2019 |

| | | | | |
|---|---|---|---|---|
| **UK Butterfly Monitoring Scheme (UKBMS)** https://catalogue.ceh.ac.uk/documents/571a676f-6c32-489b-b7ec-18dcc617a9f1 | Butterfly Conservation, UK Centre for Ecology and Hydrology (UKCEH), BTO, JNCC | Butterflies | 55 | 1976-2019 |
| **National Fish Population Database (NFPD) and Transitional/Coastal waters Data (TRaC)** Freshwater fish surveys (NFPD) - data.gov.uk | Environment Agency (EA) (data collection) Queen Mary University of London (QMUL) (analysis) | Fish | 36 | 2000-2019 |
| **Freshwater Invertebrates (BIOSYS)** https://www.data.gov.uk/dataset/3faf10d7-04bc-49e0-8377-61f75186d21d/freshwater-river-macroinvertebrate-surveys-biosys | EA (data collection) QMUL (analysis) | Freshwater invertebrates | 235 | 2013-2019 |
| **Breeding Birds Survey (BBS) Mammals** Mammal monitoring \| BTO | BTO, RSPB, JNCC | Mammals | 5 | 1995-2019 |
| **National Bat Monitoring Programme (NBMP)** https://jncc.gov.uk/our-work/national-bat-monitoring-programme/ | Bat Conservation Trust, JNCC | Mammals | 9 | 1998-2019 |
| **National Dormouse Monitoring Programme (NDMP)** https://ptes.org/campaigns/dormice/surveying-and-monitoring-hazel-dormice/national-dormouse-monitoring-programme-ndmp/ | People's Trust for Endangered Species (PTES) | Mammals (single species) | 1 | 1995-2019 |
| **Priority Moths** Home page \| Butterfly Conservation | Butterfly Conservation | Moths | 10 | 1995-2019 |
| **Rothamsted Insect Survey Light Trap** Rothamsted Insect Survey Online Database v2 - Dataset - RRES CKAN | Rothamsted Research (RRes) (collection) UKCEH (analysis) | Moths | 441 | 1970-2019 |
| **National Plant Monitoring Scheme (NPMS)** National Plant Monitoring Scheme survey data (2015-2023) - EIDC | UKCEH, Plantlife, Botanical Society of Britain and Ireland (BSBI), JNCC | Vascular plants | 180 | 2015-2019 |

*The WBI includes data from the individually mentioned breeding bird surveys

Table S2: The parameters we used to set variation in species trends for the simulated data, by taxa. We set these using the simulate_indicator function from the BRC_indicators package, which specifics error at these three levels around the underlying growth rate (mg). These are: interannual differences in the true growth rate of all species ('sdg'); interspecies differences in their true annual growth rates ('sigma'); and additional error due to observed indices differing from true indices ('SE').

We estimated values for these three parameters by running the Freeman method on the relevant dataset. We used the value 'theta' in the model output as an estimate of SE. We used the standard deviation in the posterior of logLambda means as an estimate of sdg (based on a model with a high number of knots, as sdg values initially increase with the number of knots but then plateau). We approximated a value sigma based on 'tau.spi' from the model output. Tau.spi is an estimate of precision in the distribution of species growth rates across both years and species, and therefore captures elements of both sdg and sigma. Using our estimate of sdg, we ran the Freeman method with different values of sigma until the tau.spi of the generated dataset matched the value in the relevant dataset. For the multi-taxa dataset, we estimated variation separately for each of eight groups (birds, butterflies, moths, mammals excluding bats, bats, fish, freshwater invertebrates and vascular plants), and appended simulated data to the relevant group.

Throughout, we ran the Freeman models with 5000 iterations and assumed there was no error in the indices of the first year of data.

| **Taxon** | **sdg** | **sigma** | **SE** |
|---|---|---|---|
| Birds (Bird dataset) | 0.004 | 0.08 | 0.09 |
| Lepidoptera (Lepidoptera dataset) | 0.018 | 0.12 | 0.22 |
| Birds (Multi-taxa dataset) | 0.004 | 0.08 | 0.091 |
| Butterflies (Multi-taxa dataset) | 0.040 | 0.12 | 0.207 |
| Moths (Multi-taxa dataset) | 0.031 | 0.10 | 0.262 |
| Mammals excluding bats (Multi-taxa dataset) | 0.014 | 0.04 | 0.043 |
| Bats (Multi-taxa dataset) | 0.005 | 0.02 | 0.001 |
| Fish (Multi-taxa dataset) | 0.020 | 0.20 | 0.332 |
| Freshwater invertebrates (Multi-taxa dataset) | 0.032 | 0.08 | 0.100 |
| Plants (Multi-taxa dataset) | 0.037 | 0.07 | 0.101 |

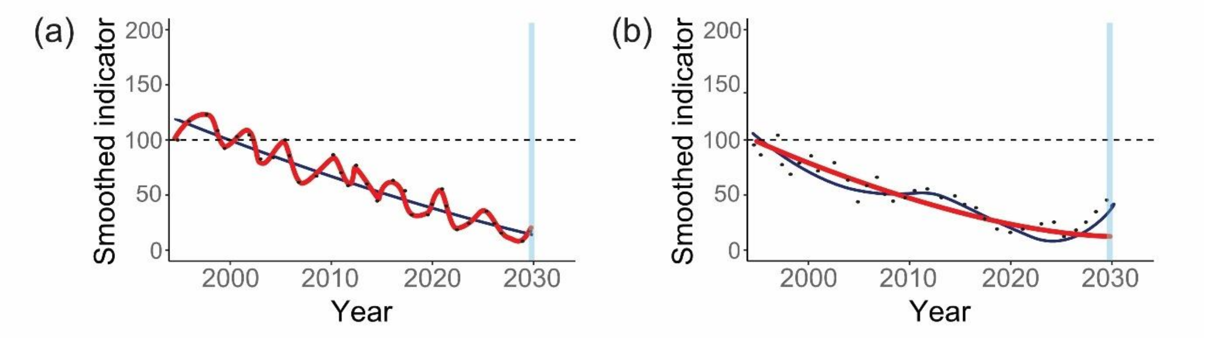


Figure S1: A hypothetical timeseries of a multi-species indicator, illustrating the risks of under- or over- smoothing. The red line is the statistically smoothed indicator, the black dots are the modelled indicator values and the blue line shows the 'true underlying' trend (i.e. a trend occurring over a relevant timescale for the target's intended aim); the target test period is indicated by the vertical shaded bar (2029-2030).

For an indicator being used to test a 'halt-the-loss' target , setting the level of smoothing involves a trade-off between two undesirable outcomes: false positive (type I) and false negative (type II) errors. An insufficiently smoothed indicator increases the risk of making a false positive error – thinking the target has been met when it has not. This occurs if the underlying trend is negative, but the indicator shows smaller fluctuations, and is therefore increasing in the test year (a). By contrast, an overly smoothed indicator increases the risk of making a false negative (type II) error – thinking the target has not been met when it has. In this case, the true underlying trend could be positive, but the indicator bends too slowly, is too smooth, to reflect the increase (b).

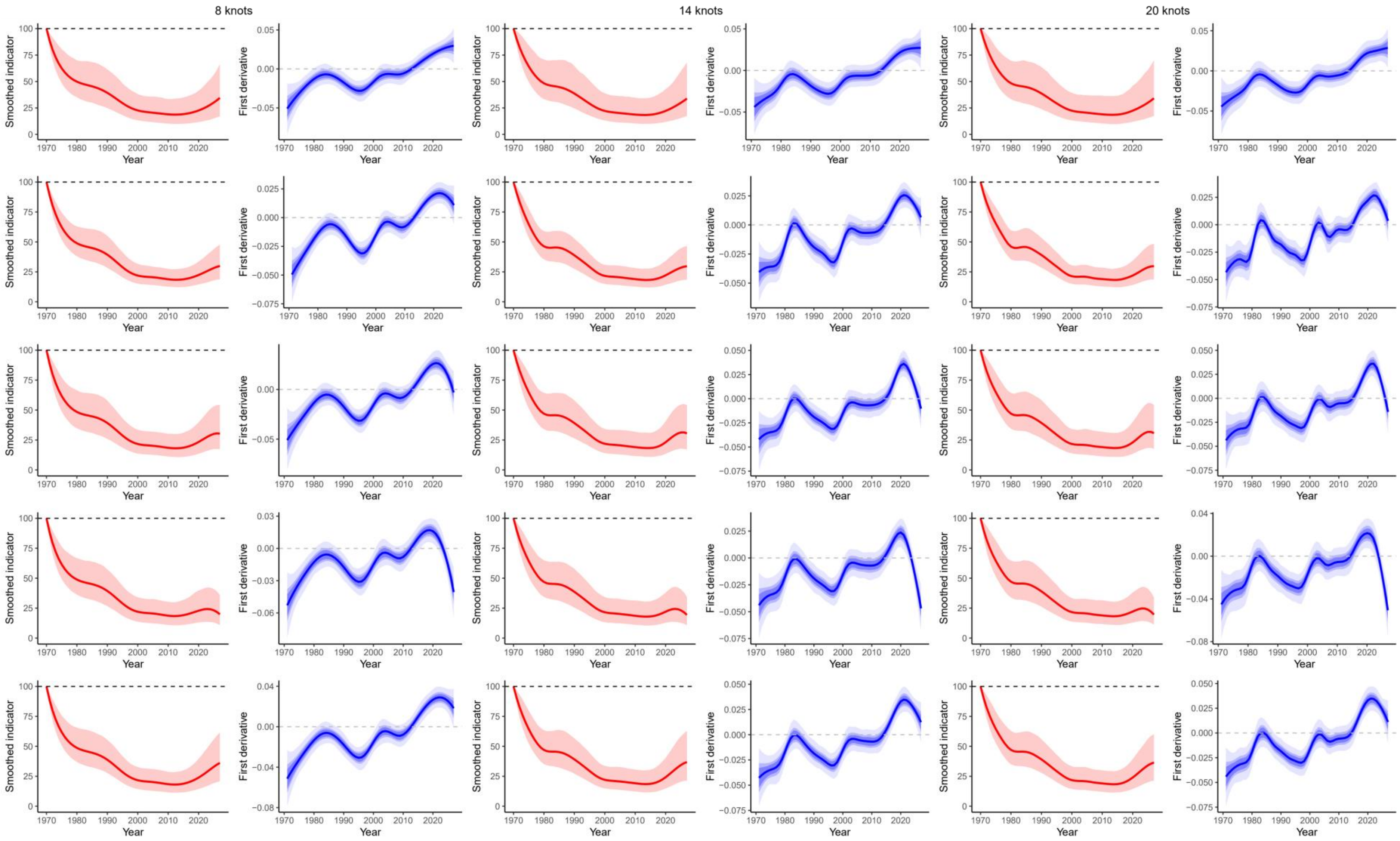


Figure S2: an example of 5 individual simulation output plots for the Lepidoptera dataset, with a simulated 3% underlying growth rate from 2019 onwards. Rows show five different runs of simulated data, with columns for 8 knots, 14 knots and 20 knots. Under each knot title is the smoothed multi-species indicator (red) and associated first derivative plot (blue). Some runs show an apparent decline over the years of simulated data (2019-2017) despite the 3% increase, due to high interannual and interspecific variability in indices.

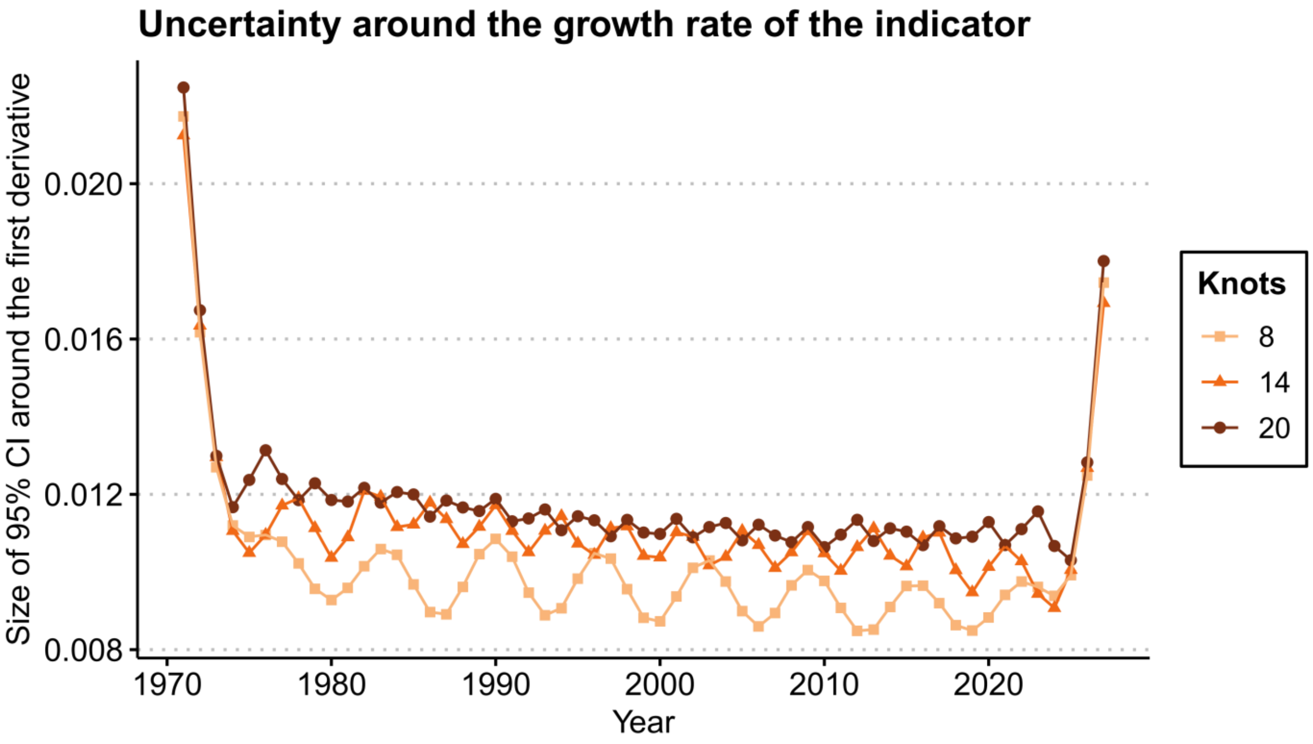


Figure S3: Average uncertainty (shown as the width of the 95% credible interval (CI)) around the estimate of the first derivative of the multi-species indicator across years. The credible interval increases in the final two years. This plot is based on the bird dataset with a simulated 1% increase; we found qualitatively similar patterns for other datasets and simulations.

This may partly explain the effect seen across all three datasets, that additional years of a particular trend initially improved the precision of the test, but not through to the final years of data. There is an outwards splaying of the credible interval at the start and end of the time series, which is a statistical characteristic of fitting a smoothed trend. If the first derivative is at 0, then the width of the CI is irrelevant for the test result (as there will be a 50% probability of being ≥ 0 regardless of the width). However, if the first derivative is above 0, more precision will be reflected in finding a higher percentage probability of being ≥ 0 (and *vice versa* if the first derivative is below zero).

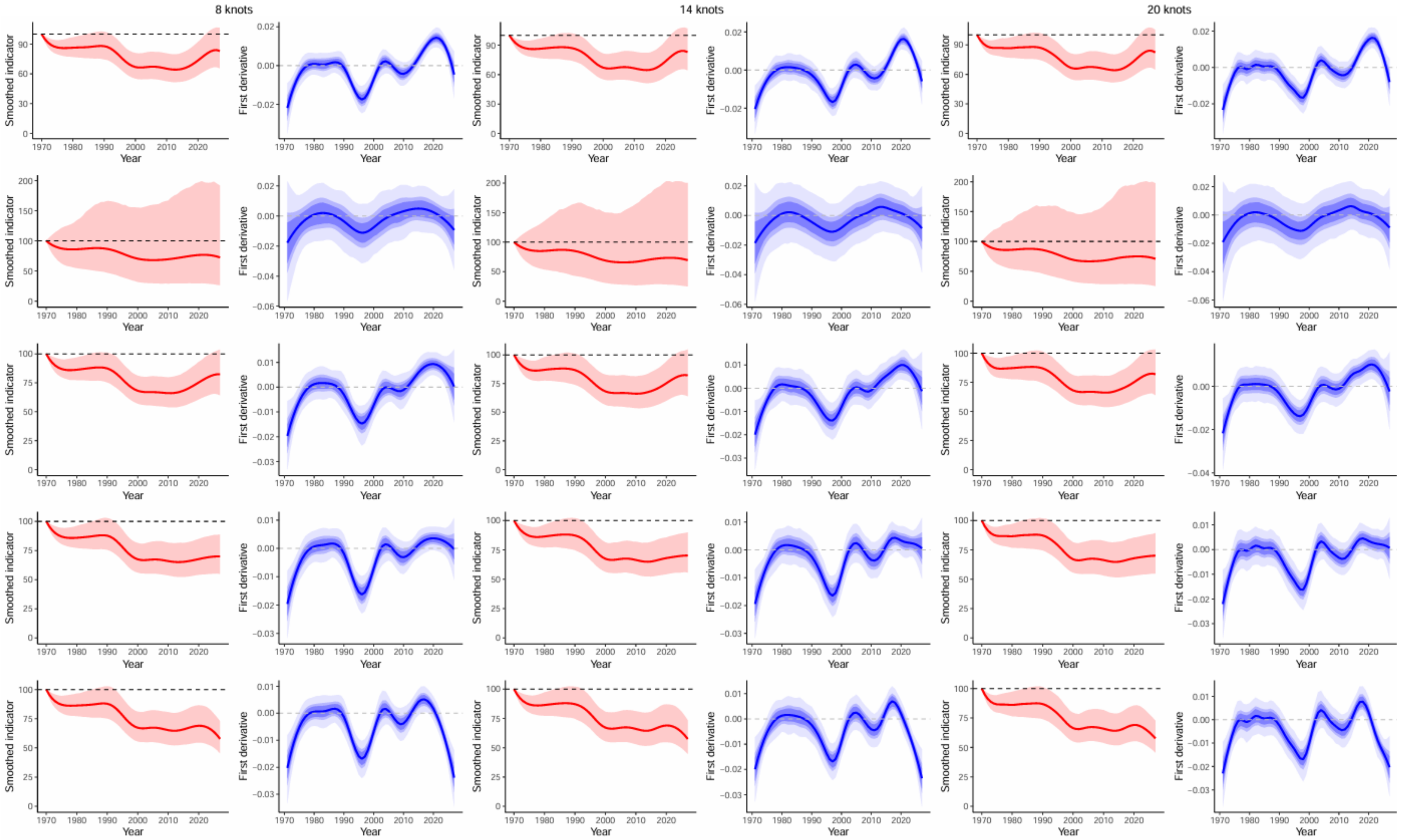
8 knots
14 knots
20 knots
Smoothed indicator
First derivative
Year

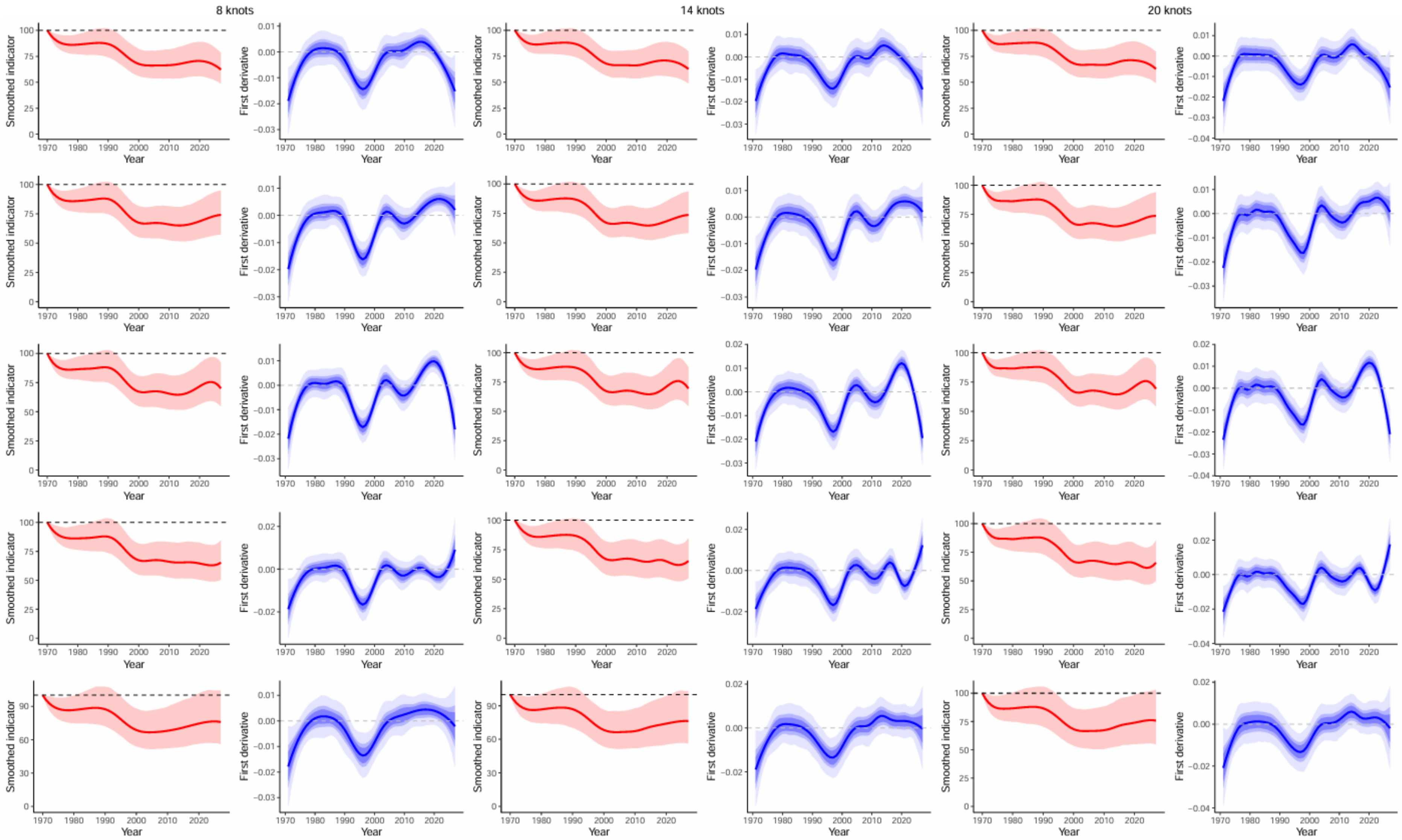
8 knots
14 knots
20 knots
Smoothed indicator
First derivative
Year

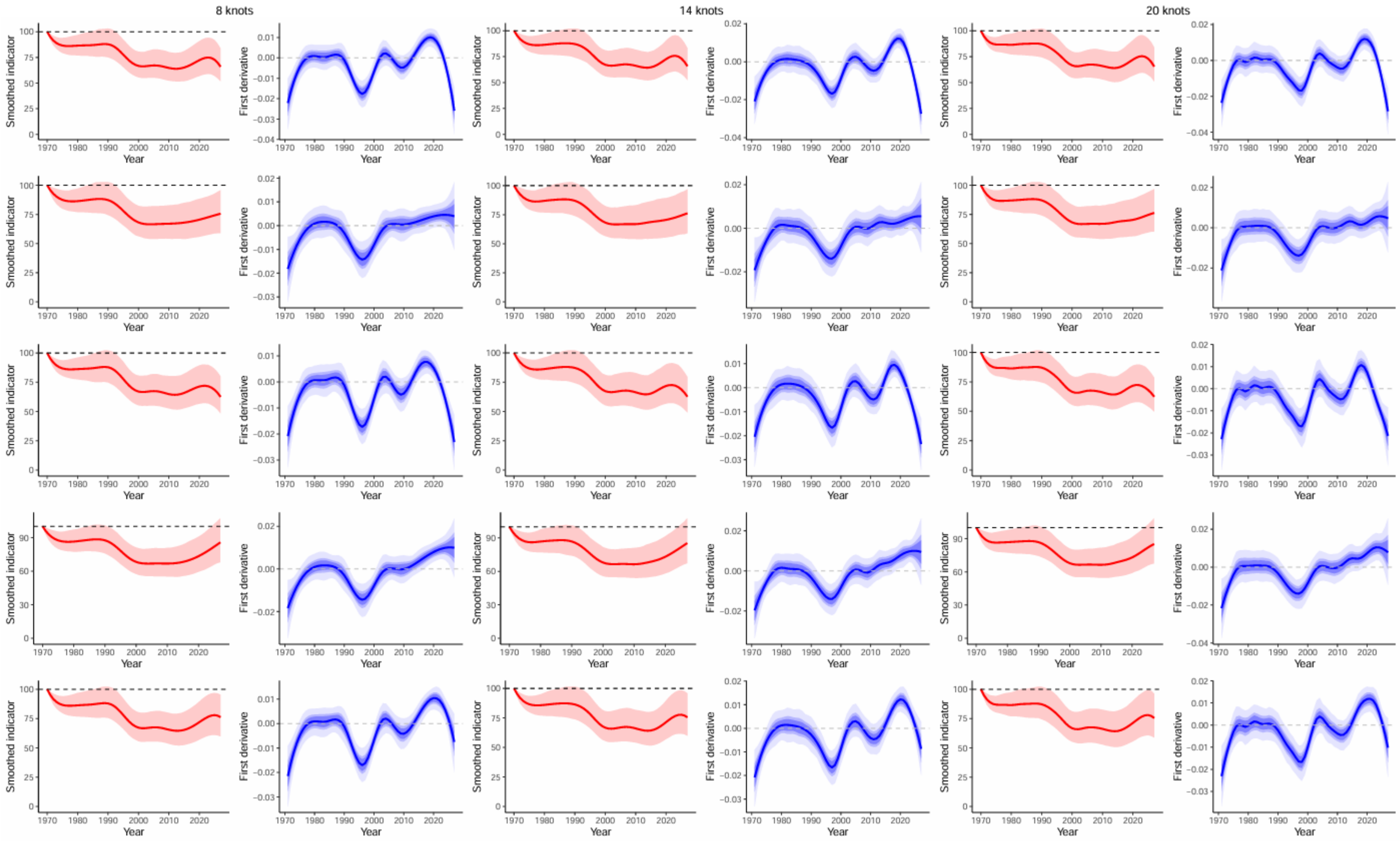
8 knots
14 knots
20 knots
Smoothed indicator
First derivative
Year

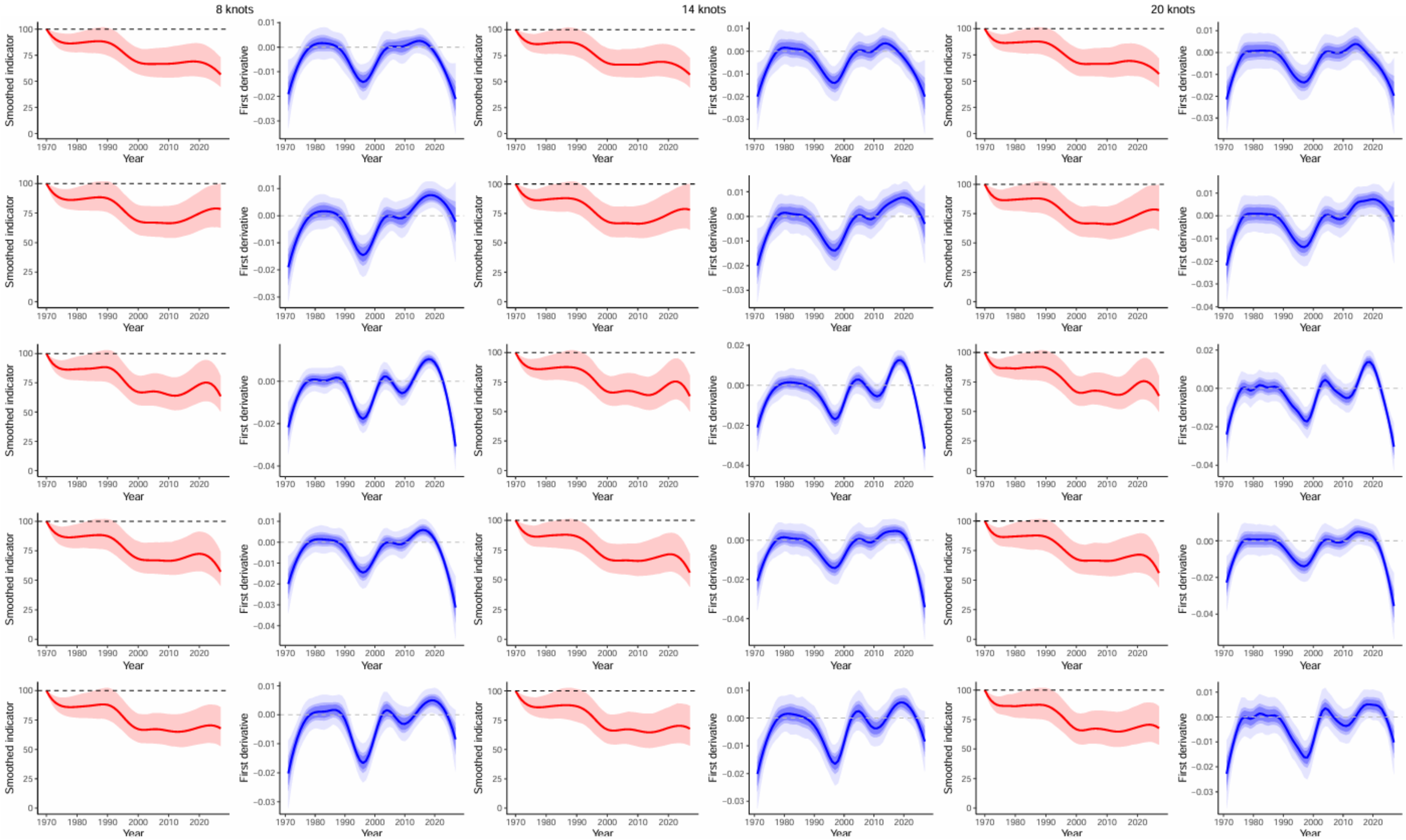
8 knots
14 knots
20 knots
Smoothed indicator
First derivative
Year

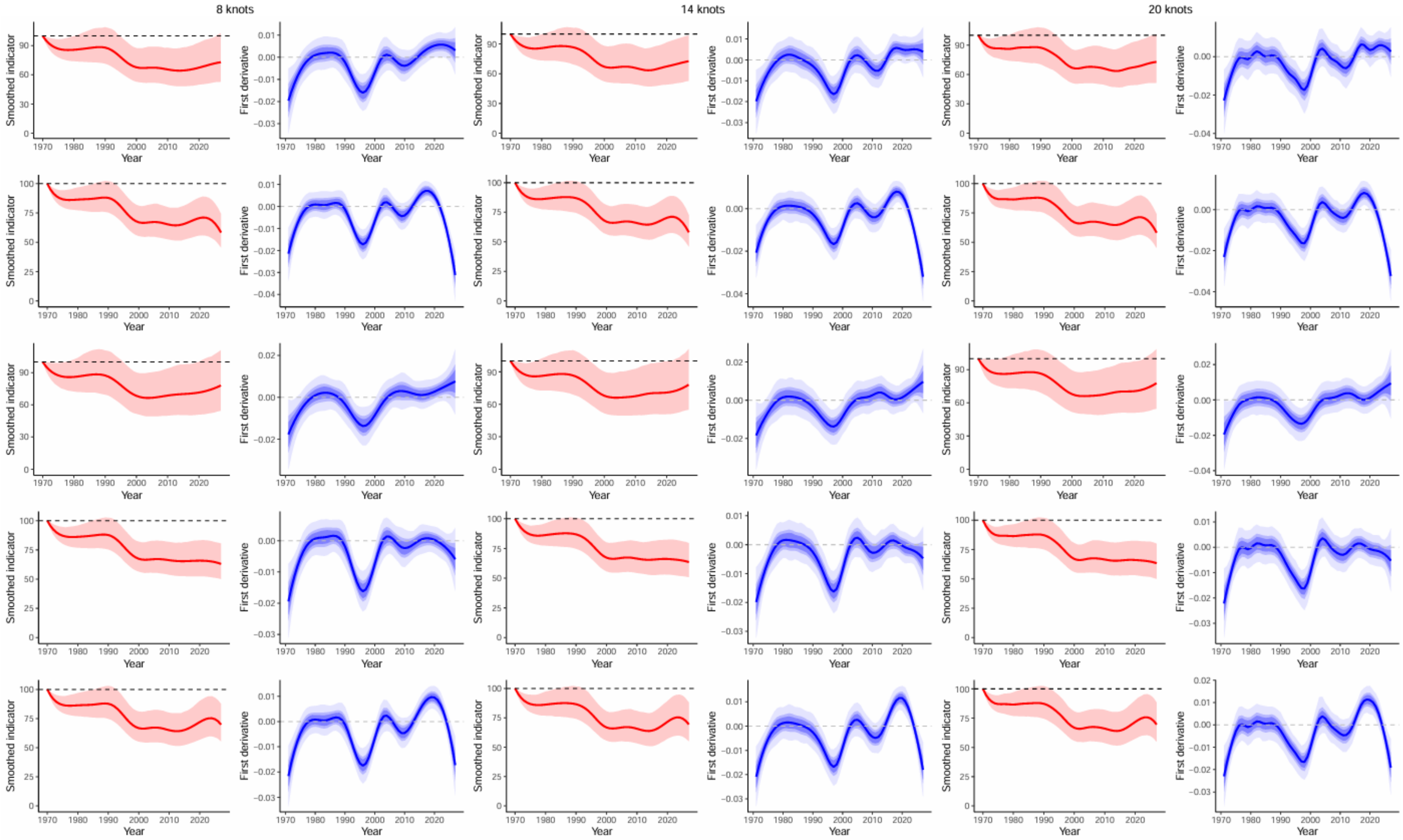

8 knots
14 knots
20 knots
Smoothed indicator
First derivative
Year

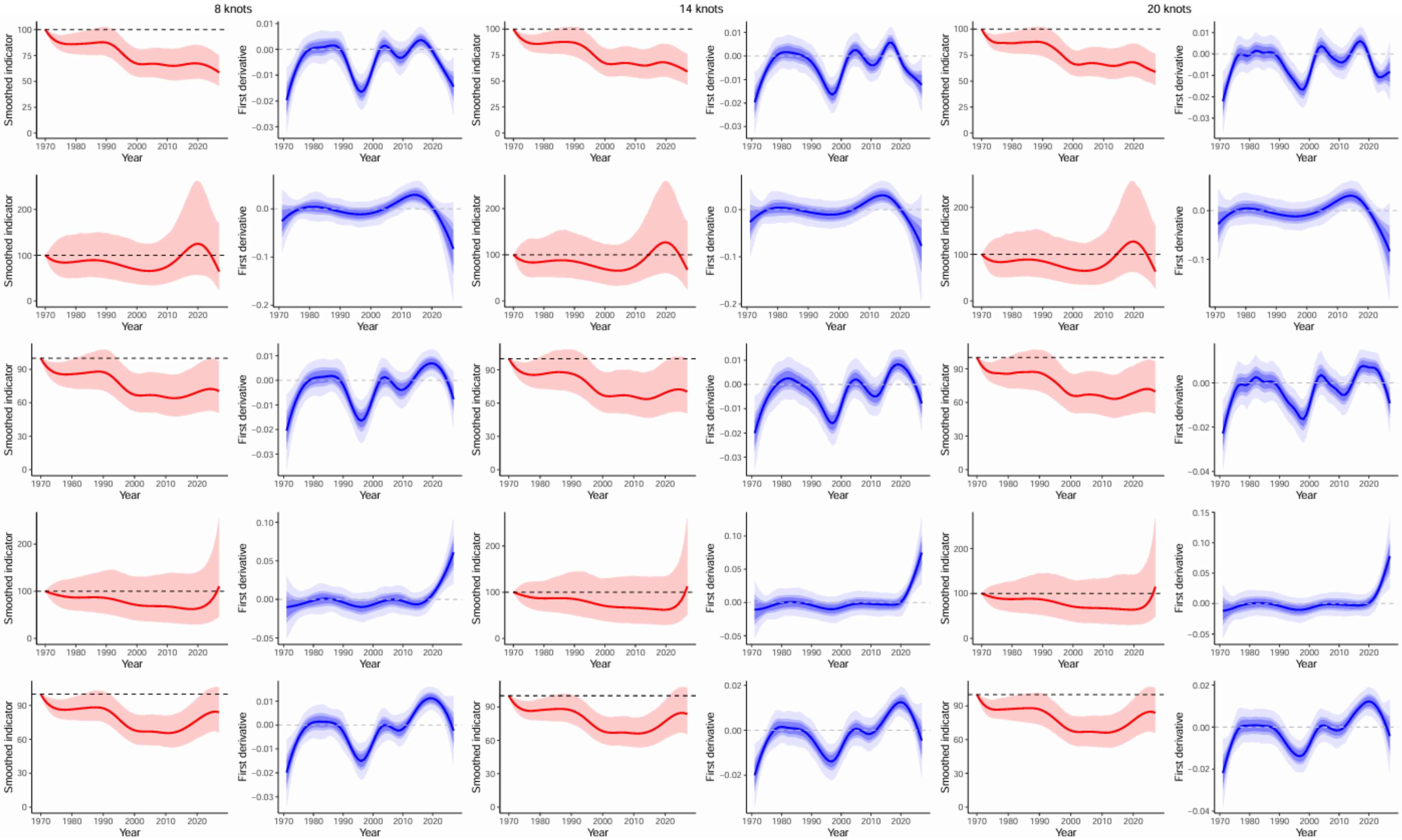
8 knots
14 knots
20 knots
Smoothed indicator
First derivative
Year

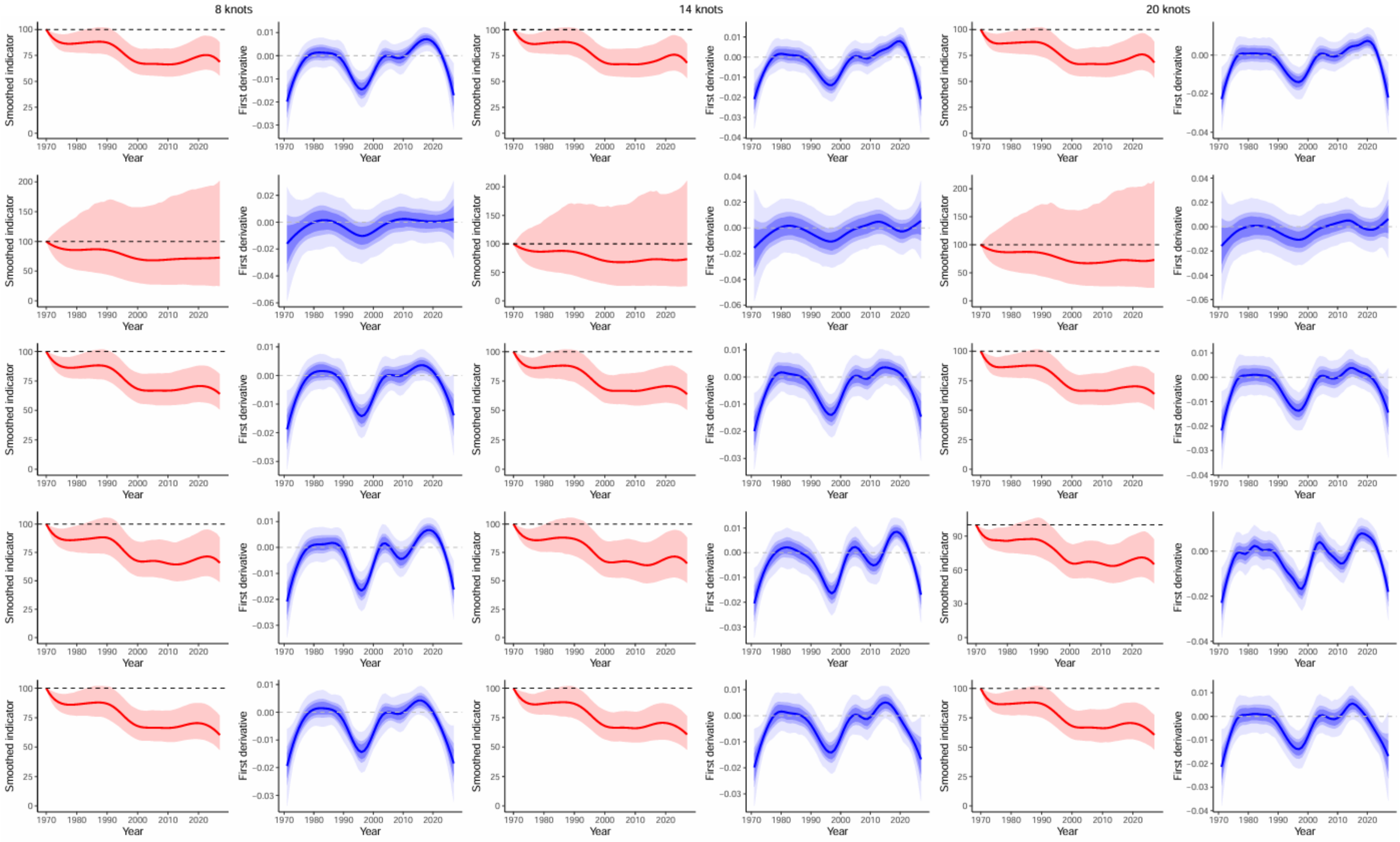
8 knots
14 knots
20 knots
Smoothed indicator
First derivative
Year

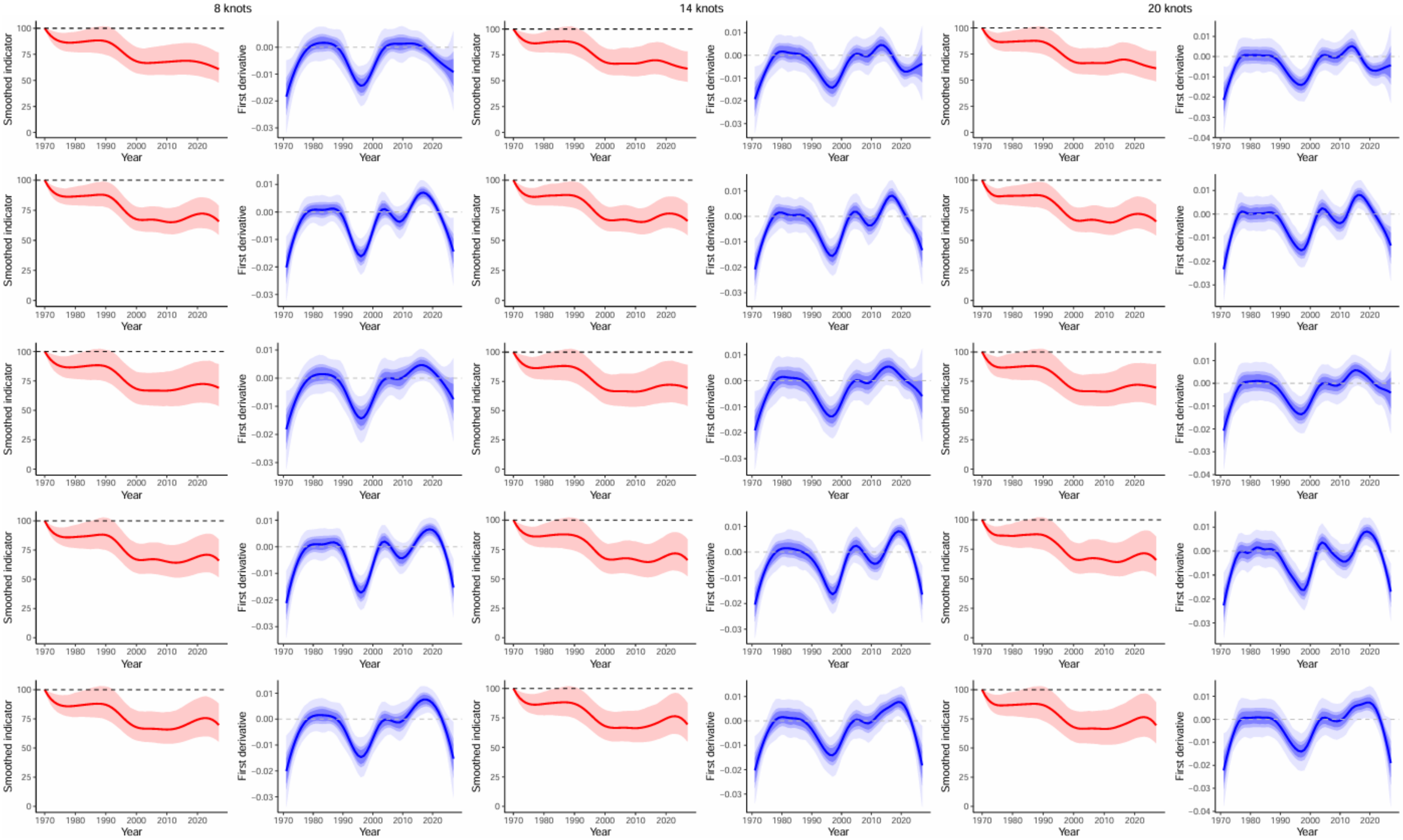
8 knots
14 knots
20 knots
Smoothed indicator
First derivative
Year

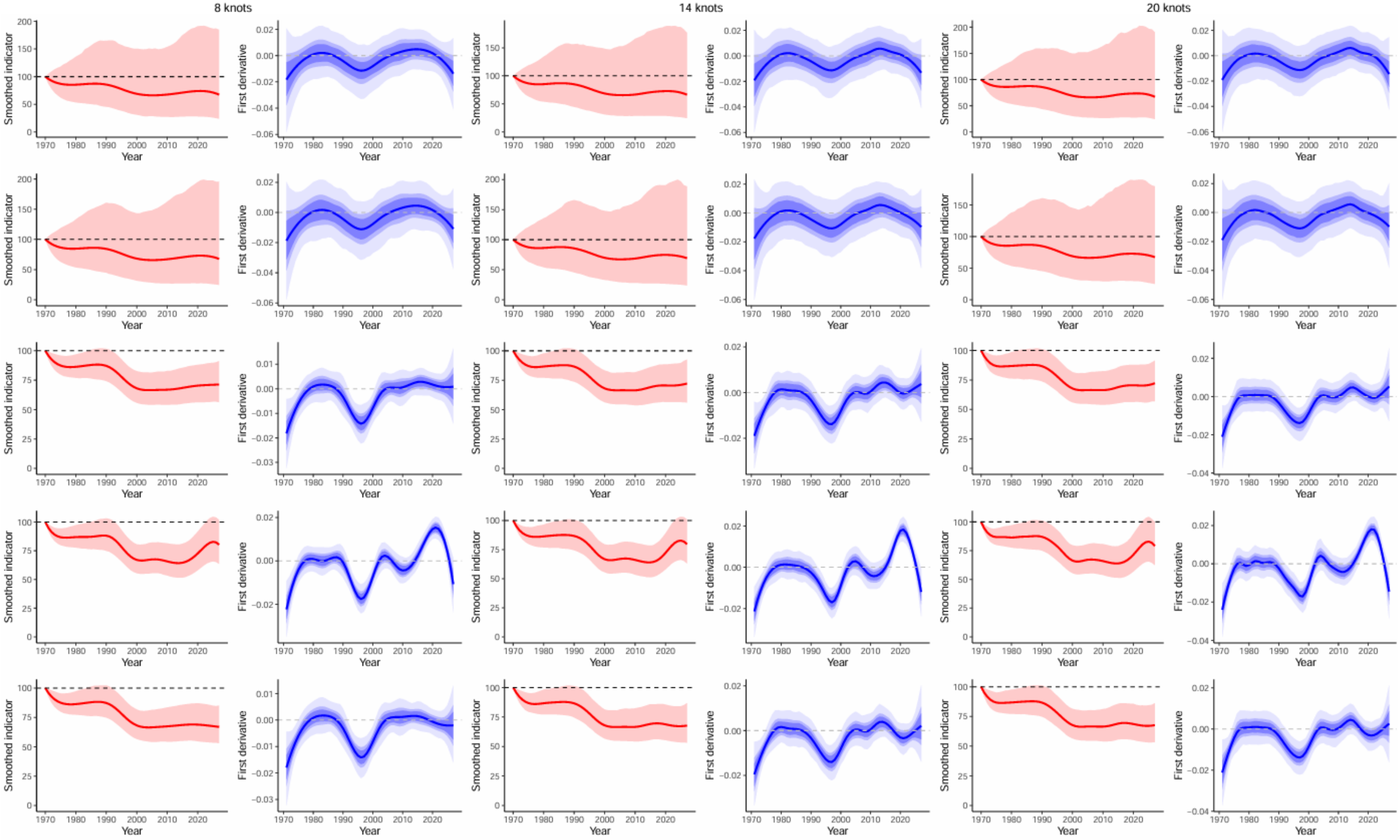
8 knots
14 knots
20 knots
Smoothed indicator
First derivative
Year

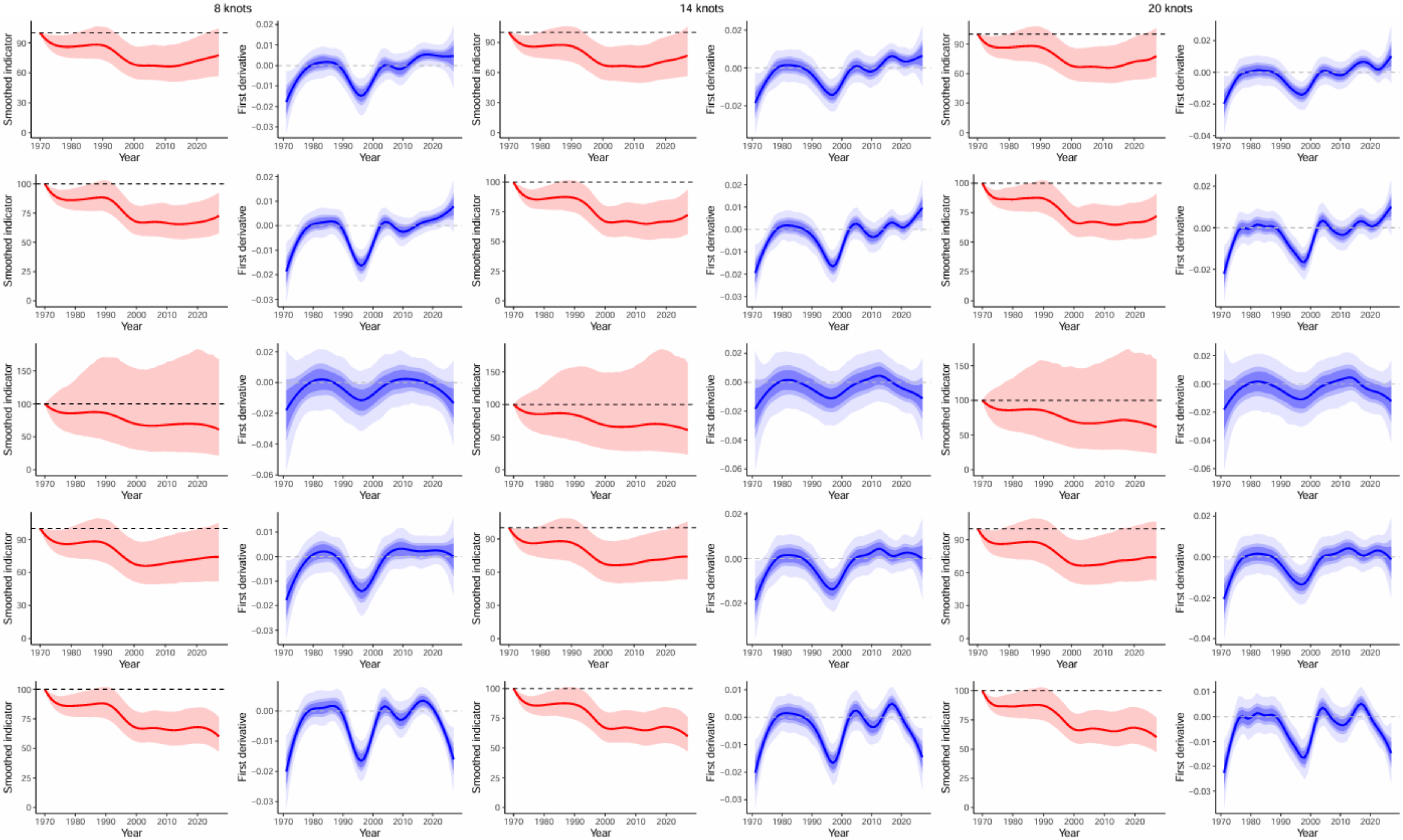
8 knots
14 knots
20 knots
Smoothed indicator
First derivative
Year

Figure S4: the full set of 50 individual simulation output plots for the multi-taxa dataset, with a simulated 0.1% underlying growth rate from 2019 onwards. Rows show fifty different runs of simulated data, with columns for 8 knots, 14 knots and 20 knots. Under each knot title is the smoothed multi-species indicator (red) and associated first derivative plot (blue). These simulations form the basis of Figure 4b (i and ii) – the average test results over all years of data, and the histogram of distributions summarising the outcome in the final year of simulated data.

This plot also shows the flaring of the credible intervals in the final years of data from the raw output (see Figure S3 for a summary figure for the bird dataset). It also shows how different values impact previous years' values in the indicator – all plots contain the same data 1970-2019, but even in this region the indicator (and test) values differ, due to smoothing meaning the indicator is fitted differently with different simulated data post-2019. It also shows the variability of simulation outcomes, with different knot numbers and across years.
